# Effectiveness and safety of moxibustion for Parkinson disease: A protocol for systematic review and meta-analysis

**DOI:** 10.1097/MD.0000000000026256

**Published:** 2021-06-11

**Authors:** Yonghui Hou, Baile Ning, Yamin Liu, Ying Liu, Wenbin Fu, Zehuai Wen

**Affiliations:** a Shijiazhuang People's Hospital, Shijiazhuang, Hebei; b The Second Affiliated Hospital of Guangzhou University of Chinese Medicine; c Situ Ling Studio of Lingnan Acupuncture School; d Famous Doctor's Studio of Academician Shi Xuemin; e Shenzhen Bao’an Research Center for Acupuncture and Moxibustion, Shenzhen; f National Center for Design Measurement and Evaluation in Clinical Research, Guangzhou University of Chinese Medicine, Guangzhou, Guangdong, China.

**Keywords:** meta-analysis, moxibustion, Parkinson disease, protocol

## Abstract

**Background:**

Parkinson disease (PD) is a common neurodegenerative disease among middle-aged and elderly people. Clinically, it is a movement disorder characterized mainly by static tremors, kinesia, myotonia, and postural balance disorder. In recent years, an increasing number of clinical reports on moxibustion therapy for PD have been published. Despite this, no systematic review of moxibustion therapy for PD has been undertaken.

**Methods:**

Two reviewers will search the following 7 English and Chinese databases online: the Cochrane Library; PubMed; EMBASE; the China National Knowledge Infrastructure; the Wan Fang databases; the China Science and Technology Journal Database; and the Chinese Biomedical Literature Database. Reviewers will search each electronic database for studies published from journal inception to May 2021. Two reviewers will independently conduct clinical study inclusion, data extraction, and risk bias assessment. Any differences in the above process will be resolved through discussion with a third reviewer. If the data are sufficient, RevMan software 5.3 (Cochrane Community, London, UK) will be used for the meta-analysis of the extracted data.

**Results:**

In this systematic review, the effectiveness and safety of moxibustion therapy in PD treatment will be evaluated.

**Conclusion:**

This systematic review may provide further evidence to encourage clinicians to use moxibustion in the treatment of PD.

**INPLASY registration number:**

INPLASY202140097

## 1 Introduction

### 1.1 Description of the condition

Parkinson disease (PD) is a common neurodegenerative disease among middle-aged and elderly people. Clinically, it is a movement disorder characterized mainly by static tremors, kinesia, myotonia, and postural balance disorder.^[1]^ It may also be accompanied by a number of non-motor symptoms, including depression, anxiety, sleep disorders, constipation, and hyposmia.^[2]^ The prevalence of PD is 1% in people aged over 60 years and over 4% in people aged over 80 years.^[3]^ It is anticipated that the number of people with PD in China will increase from 1.99 million in 2005 to 5 million in 2030, accounting for almost half of the global number of PD patients.^[4]^ The main pathological changes in PD involve the gradual degeneration of dopaminergic neurons of the substantia nigra and the formation of Lewy bodies, resulting in a reduction of dopamine transmitters in the striatum.^[5]^ As the disease progresses, PD lowers the quality of life of patients and their caregivers and severely increases the economic burden on families and society.^[6]^

Treatment methods for PD include drug therapy, surgical therapy, botulinum toxin therapy, exercise therapy, psychological intervention, and nursing care. Drug therapy, such as oral dopamine preparation, is the treatment of choice, and the main treatment in the whole treatment process.^[7]^ However, although the symptoms of patients can be controlled within a certain period, the effects do not last, and adverse reactions such as impulse control disorders, hallucinations, or the “on-off” phenomenon may occur.^[8]^ Therefore, many PD patients in China seek moxibustion as a complementary and alternative therapy.

### 1.2 Description of the intervention

Moxibustion is an external treatment method that uses ignited moxa floss to stimulate the meridians and acupoints in order to prevent and cure diseases. It is an important external therapy in traditional Chinese medicine (TCM), which was first recorded in Zuo Zhuan in 581 BC. Moxibustion involves moxa stick moxibustion, moxa cone moxibustion, needle warming moxibustion, and moxa burner moxibustion.

Moxibustion warms the body, dispels cold, promotes blood circulation, regulates energy metabolism, and alleviates pain.^[9]^ In clinical practice, the proper indications for moxibustion therapy include malposition, diarrhea, urinary incontinence, knee osteoarthritis, stroke, asthma, soft tissue injury, weakness, and aging-related problems.^[10–12]^

### 1.3 How the intervention might work

The mechanism of moxibustion in the treatment of treating PD remains unclear. The possible mechanism of moxibustion may be mainly related to the thermal effect, radiation effect, and pharmacological effects of the moxa stick and its combustion products.^[10]^ Based on the theory of TCM, moxibustion is considered to reconcile qi and the blood, enhance physical fitness, and excrete the pathogenesis by means of warming.^[13]^

### 1.4 Why is it essential to perform this review?

In TCM, PD belongs to the category of fibrillation syndrome. As early as 1146 AD, Bian Que Xin Shu recorded moxibustion at the Guanyuan acupoint for the treatment of PD. In recent years, there have been an increasing number of clinical reports on moxibustion therapy for PD.^[14–16]^ However, there is no systematic review of moxibustion therapy for PD. Therefore, it is very important to perform this review.

### 1.5 Objectives

This review proposes to appraise the effectiveness and safety of moxibustion for PD, which may provide a complementary and alternative therapy for PD.

## 2 Methods

### 2.1 Study registration

This study was prospectively recorded in the International Platform of Registered Systematic Review and Meta-Analysis Protocols (registration number INPLASY202140097) on April 18, 2021. This protocol follows the statement guidelines of the preferred reporting items for systematic reviews and meta-analysis protocols (PRISMA-P).^[17]^

### 2.2 Inclusion and exclusion criteria

#### 2.2.1 Study types

All randomized controlled trials (RCTs) on moxibustion for PD published in Chinese and English will be included. Others such as non-RCTs, case reports, review studies, animal experiments, and laboratory studies will be excluded.

#### 2.2.2 Types of participants

We will recruit patients with a clinical diagnosis of PD regardless of sex, age, race, or severity.

#### 2.2.3 Types of intervention

The control group will receive western medicine. The experimental group will receive moxibustion alone or in combination with western medicine, regardless of treatment duration and dosage.

#### 2.2.4 Types of outcome measures

##### 2.2.4.1 Primary outcomes

The primary outcomes will be the unified Parkinson disease and rating scale results and the total effective rate.

##### 2.2.4.2 Secondary outcomes

The secondary outcomes will be the Quality of Life Questionnaire results and adverse events.

### 2.3 Search strategy

Two reviewers will search the following 7 English and Chinese databases online: the Cochrane Library; PubMed; EMBASE; China National Knowledge Infrastructure; the Wan Fang databases; the China Science and Technology Journal Database; and the Chinese Biomedical Literature Database. Reviewers will search each electronic database for studies published from journal inception to May 2021. The main subject terms searched were: “moxibustion”; “Parkinson”; “parkinsonism”; and “Parkinson's disease.” The search strategy for PubMed is displayed in Table 1. Modified search strategies will be adopted for the other electronic databases.

**Table 1:** Search strategy for the PubMed database.

Number	Search terms
#1	“moxibustion”[Mesh]
#2	“moxibustion”[Ti/Ab]
#3	#1or#2
#4	“Parkinson disease”[Mesh] OR “Parkinson”[Mesh]OR “parkinsonism”[Mesh]
#5	“Parkinson disease”[Ti/Ab] OR “Parkinson”[Ti/Ab]OR “parkinsonism”[Ti/Ab]
#6	#4or#5
#7	“Randomized controlled trial” [MeSH] or “controlled clinical trial” [MeSH]
#8	“Randomized controlled trial” [Ti/Ab] or “randomized” [Ti/Ab] or “clinical trial” [Ti/Ab]
#9	#7 or #8
#10	#3 and #6 and #9

### 2.4 Study selection

**Figure 1:**
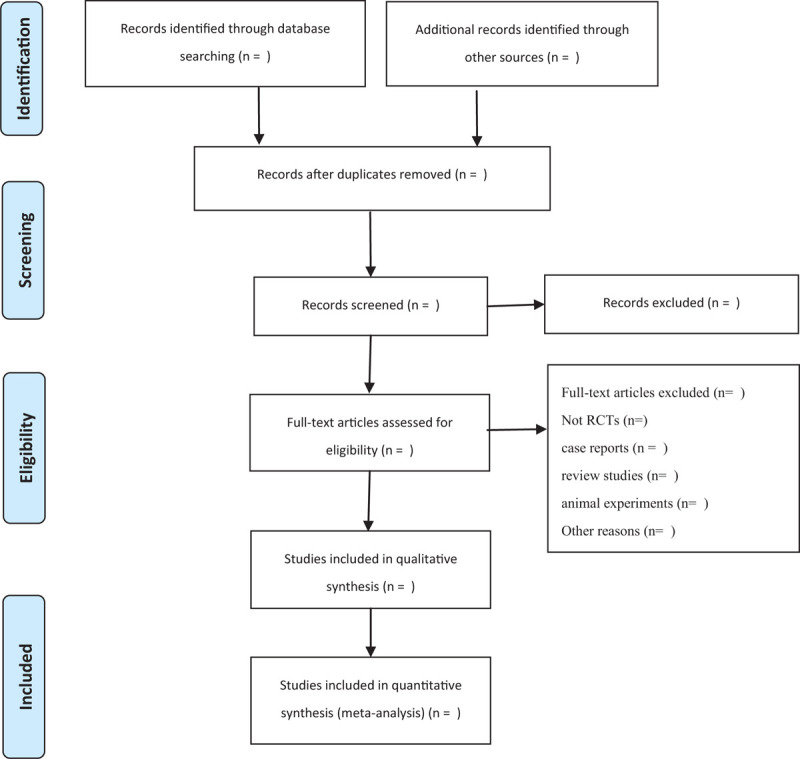
PRISMA flow chart. PRISMA = preferred reporting items for systematic reviews and meta-analysis.

After the search is complete, we will enter all retrieved studies into NoteExpress 3.2 (https://noteexpress1.software.informer.com/3.2/) to remove duplicates. Two reviewers will independently screen all related studies by titles, abstracts, keywords, and full texts if required. Subsequently, 2 reviewers will read the remaining documents carefully for further filtration. Furthermore, a third reviewer will decide upon a study when a dispute arises. The detailed selection process will be presented as a PRISMA flowchart (Fig. 1).

### 2.5 Data extraction and management

A uniform data form will be used by 2 reviewers to extract data independently. The form covers the title, first author, publication year, journal title, country, study design, methodological quality, patient characteristics, control intervention, experimental intervention, duration of intervention, outcomes, adverse events, and follow-up. If the information needed is not available, reviewers will try to communicate with the corresponding authors. Any disagreement will be resolved by negotiating with the third reviewer.

### 2.6 Assessment of the risk of bias in included studies

Two reviewers will use the Cochrane risk of bias assessment tool to independently evaluate the risk of bias in the chosen studies. This tool covers:

(1) Randomized sequence generation;
(2) Allocation sequence concealment;
(3) Blinding of participants and personnel;
(4) Blinding of outcome assessment;
(5) Incomplete outcome data;
(6) Selective outcome reporting;
(7) Other biases (e.g., conflict of interest and intention-to-treat).

Each item will be categorized as “high risk,” “unclear risk,” or “low risk.” Any disagreement will be settled by negotiating with the third reviewer.

### 2.7 Data synthesis and analysis

#### 2.7.1 Measurements of treatment effect

The mean difference (MD) with 95% confidence interval (95% CI) will be assumed to measure the effect for continuous variables. Risk ratios (RR) with 95% CIs will be adopted to measure the curative effect for dichotomous variables.

#### 2.7.2 Assessment of heterogeneity

Heterogeneity refers to any variation among studies in a systematic evaluation.^[18]^

Heterogeneity will be calculated with the chi-squared test. If *I*^2^ < 50% and *P* > .1%, this will indicate that the heterogeneity among the RCTs is low, and the fixed-effect model will be adopted. If *I*^2^ > 50% and *P* < .1%, this will suggest that the heterogeneity among the RCTs is high, and the random effects model will be applied.

#### 2.7.3 Data synthesis

If it is reasonable to carry out a meta-analysis, RevMan V.5.3 software (Cochrane Community, London, UK) will be applied for data synthesis. If quantitative analysis is not appropriate, a systematic narrative synthesis or qualitative analysis will be employed to illustrate the findings of the included studies.^[19]^

#### 2.7.4 Subgroup analysis

If there are enough studies and available information, we will implement subgroup analyses according to patient condition, intervention measures, and outcome measurements.

#### 2.7.5 Sensitivity analysis

To test the stability and reliability of the meta-analysis, a sensitivity analysis will be conducted considering the following aspects:

(1) Replacing fixed effects models with random effects models, and vice versa.
(2) Excluding studies of low quality or with missing data.

#### 2.7.6 Assessment of reporting biases

If sufficient studies (>10 studies) are involved, funnel plots of RevMan V.5.3 software (Cochrane Community) will be adopted to appraise potential publication bias. Otherwise, STATA 13.0 software (Stata, College Station, TX) will be employed to perform Egger test.

#### 2.7.7 Grading the quality of evidence

The Grading of Recommendations Assessment, Development, and Evaluation^[20]^ will be used to appraise the quality of evidence for outcomes, covering the 5 fields (limitations of study, inconsistency, indirectness, imprecision, and reporting biases). The assessments will be ranked as “very low,” “low,” “moderate,” and “high.”

#### 2.7.8 Ethics and dissemination

Because this is a review, which does not involve individuals or animals, ethical approval will not be required. Once the results of this review are concluded, they will be submitted to a peer-reviewed journal.

## 3 Discussion

PD is the second most prevalent progressive neurodegenerative disease subsequent to Alzheimer disease, and it causes both motor and nonmotor symptoms.^[21]^ Furthermore, the growing incidence of PD also places a large economic burden on patients and on society. The mechanism of moxibustion as a non-drug treatment for PD remains unclear, despite the fact that its efficacy has been shown with almost no side effects and few adverse reactions. However, no meta-analysis and systematic review on the effectiveness and safety of moxibustion in treating patients with PD has been published. Therefore, we will carry out a systematic review to explore the effectiveness and safety of moxibustion for PD. We expect that this systematic review will provide more evidence for clinical staff and future researchers.

## Author contributions

**Conceptualization:** Ying Liu, Wenbin Fu.

**Data curation:** Yonghui Hou, Yamin Liu.

**Formal analysis:** Yonghui Hou, Yamin Liu.

**Funding acquisition:** Baile Ning, Wenbin Fu.

**Software:** Yonghui Hou.

**Supervision:** Zehuai Wen.

**Writing – original draft:** Yonghui Hou.

**Writing – review & editing:** Yonghui Hou, Baile Ning.
